# Engrailed-2 and inflammation convergently and independently impinge on cerebellar Purkinje cell differentiation

**DOI:** 10.1186/s12974-024-03301-6

**Published:** 2024-11-28

**Authors:** Mohammed Bahaaeldin, Carolin Bülte, Fabienne Luelsberg, Sujeet Kumar, Joachim Kappler, Christof Völker, Karl Schilling, Stephan L. Baader

**Affiliations:** 1 Institute of Anatomy, Anatomy and Cell Biology, University of Bonn, Nussallee 10, 53125 Bonn, Germany; 2 National Reference Laboratory for Tuberculosis, ICMR-RMRC, Bhubaneswar, Odisha India; 3 Institute of Biochemistry and Molecular Biology, University of Bonn, Nussallee 11, 53125 Bonn, Germany

**Keywords:** Cerebellum, Purkinje cell differentiation, Inflammation, Engrailed, Slice culture, LPS, Autism, Microglia, Tumor necrosis factor alpha

## Abstract

Autism spectrum disorders (ASD) have a complex pathogenesis thought to include both genetic and extrinsic factors. Among the latter, inflammation of the developing brain has recently gained growing attention. However, how genetic predisposition and inflammation might converge to precipitate autistic behavior remains elusive. Cerebellar structure and function are well known to be affected in autism. We therefore used cerebellar slice cultures to probe whether inflammatory stimulation and (over)expression of the autism susceptibility gene Engrailed-2 interact in shaping differentiation of Purkinje cells, key organizers of cerebellar histogenesis and function. We show that lipopolysaccharide treatment reduces Purkinje cell dendritogenesis and that this effect is enhanced by over-expression of Engrailed-2 in these cells. The effects of lipopolysaccharide can be blocked by inhibiting microglia proliferation and also by blocking tumor necrosis factor alpha receptor signaling, suggesting microglia and tumor necrosis factor alpha are major players in this scenario. These findings identify Purkinje cells as a potential integrator of genetic and environmental signals that lead to an autism-associated morphology.

**Supplementary Information:**

The online version contains supplementary material available at 10.1186/s12974-024-03301-6.

## Background

Autism is a developmental disorder that becomes typically evident in children before their third birthday. Individuals affected often display limited social interactions, stereotyped behavior, and speech disorders. Various factors, both extrinsic and genetic, are discussed as potential causes, likely operating cooperatively through multiple convergent pathways [1]. In recent years, inflammation during pregnancy has emerged as a significant condition affecting brain development [2–4]. Inflammatory mediators are believed to disrupt neuron development and alter synaptic connections [5].

Autism pathology affects various areas of the brain, notably the limbic brain [6, 7], and, a growing body of literature suggests a pathomechanistic involvement of the cerebellum in autism [6, 8–11]. This perspective accounts for the increasingly acknowledged role of the cerebellum in cognition and emotion [12–14], and it is supported by evidence showing consistent cerebellar morphological alterations in autistic individuals [6, 9, 15].

The adult cerebellar cortex consists of three layers, with millions of granule cells forming the innermost layer. These glutamatergic interneurons send parallel fibers toward the dendrites of Purkinje cells (PCs) in the outermost molecular layer. Between these two layers, PC somata are aligned as a monolayer. PCs receive synaptic input not only from granule cells, but also from extracerebellar climbing fibers, a set of cerebellar inhibitory interneurons, and neighboring PCs [16]. In mice, the formation of the cerebellar cortex begins around embryonic day 8 (E8) when the isthmic organizer induces the cerebellar anlage. PC precursors proliferate until approximately E13; they then migrate to form an initially multilayered band of cells close to the surface of the cerebellar anlage [17, 18]. Subsequently, PCs develop their characteristic dendritic arborization between birth (E19/P0) and postnatal day 5 (P5). Dendritic development commences with the elaboration of multiple dendrites from the soma. These initial dendrites subsequently regress, and a single main stem dendrite emerges and divides in a dichotomized pattern, forming a mature dendritic arbor around postnatal day 17 [19, 20].

Numerous genetic and extrinsic factors are known to regulate the complex interplay between developing cerebellar cells. Among the genes identified as critical for cerebellar development, the homeodomain protein Engrailed-2 (EN2) is of particular interest in the present context. Firstly, it has been described as a susceptibility locus for Autism Spectrum Disorders (ASD) [21–25] and is scored in the SFARI database as a strong candidate gene (https://gene.sfari.org/). Secondly, EN2 misexpression has been shown to affect several aspects of cerebellar morphogenesis that may relate to the aberrant cerebellar morphology observed in autistic individuals [26, 27]. Thus, EN2 governs cell survival and fate determination in early cerebellar and midbrain development [28, 29]. Moreover, in mice, prolonged expression of EN2 during late embryogenesis and early postnatal development causes PC death, slows PC dendritic development, and disrupts the proper organization of the cerebellar cortex into sharply defined, PC-based modules [30–32]. This phenotype is reminiscent of the cerebellar morphology previously described in ASD patients: a decrease in cell size and number, altered neuronal differentiation, and changes in synapse formation [33–35].

Intriguingly, the most relevant ASD-associated variant of human *EN2* is characterized by a single nucleotide polymorphism that enhances EN2 expression in vitro [36]. Consistently, *EN2* gene levels were found to be increased in the brains of ASD patients [37]. This suggests that L7En-2 transgenic mice might be a suitable model to probe the morphological development of the cerebellum as it relates to ASD. In these mice, the physiologic perinatal downregulation of EN2 in cerebellar PCs is countered by the upregulation of an L7-driven EN2 transgene during a time window [30] equivalent to the period when ASD symptoms become apparent in humans [38].

As indicated above, besides genetic predisposition as represented by mutant EN2, prenatal inflammation may also increase the risk of developing autism [39, 40]. Therefore, in this study, we utilized slice cultures established from early postnatal L7En-2 transgenic mice to investigate whether and how this genetic alteration interacts with inflammatory stimulation during a critical phase of PC development. Slice cultures are a well-established model wherein PCs recapitulate basic steps of neuronal development and can be easily manipulated pharmacologically [32]. Our results demonstrate that both protracted PC-specific expression of the autism susceptibility gene *En2* and inflammation independently affect PC differentiation. Their effects thus appear to be additive. The inflammatory effects, but not those of EN2 overexpression, were dependent on microglial activation and involved tumor necrosis factor alpha signaling.

## Materials and methods

### Animal husbandry

The L7En-2 mice utilized in this study were generated by mating wildtype FVB/N mice with heterozygous L7En-2 animals [30]. These mice were maintained on a pure FVB/N background throughout the study. For experiments not involving manipulation of EN2 expression, C57Bl6/J mice were used. All animal handling procedures strictly adhered to local governmental and institutional animal care regulations, and mice had ad libitum access to food and water. Age-matched genotypes were obtained by overnight mating, with successful mating confirmed by mating plugs. The day of birth was designated as postnatal day 0 (P0). At least two pairs of littermates were selected for each experiment. Genotyping was performed as previously described using L7En-2 specific primers [30].

### Preparation of an Engrailed-2 specific rabbit antiserum

The full-length mouse Engrailed-2 (*En2*) sequence was amplified from cerebellar mouse tissue via PCR and subsequently cloned into a pQE80L vector containing HIS/GST tag sequences. The EN2 protein was then produced in BL-21 bacteria and purified by passage over a GST affinity column. Following purification, the extract was dialyzed against PBS and administered to rabbits by three subsequent subcutaneous injections (Pineda Antikörper Service, Berlin, Germany). The titer of the resulting antibody was assessed using Western blotting. After the third boost, rabbits were bled, and the EN2 antibody was purified from rabbit blood serum using GST and mannose-binding protein (MBP) affinity chromatography [41]. To generate the bait for MBP affinity chromatography, *En2* was cloned into the plasmid pRK793 (Addgene), in which the coding region for *En2* was fused with a C-terminal His-tag and an N-terminal MBP-tag. Subsequently, MBP-EN2-HIS6 protein was expressed in BL-21 bacteria and purified through amylose resin chromatography, followed by Nickel agarose chromatography and dialysis against PBS. Affinity chromatography was carried out according to the manufacturer's instructions (NEB, Frankfurt, Germany). The specificity of the antiserum was verified using Western blotting and immunohistochemistry on material derived from wildtype, L7En-2 transgenic and En2^ntd/ntd^ knockout mice [42] (see Suppl. Figure 1).

### Cerebellar slice cultures

Cerebellar slice cultures were prepared from newborn (P0) and six-day-old pups (P6). Mice were euthanized via decapitation, and cerebellar slices were meticulously prepared following the protocol outlined by Jankowski et al. [32], with slight adjustments. Brains were aseptically dissected from the skull and promptly collected and submerged in ice-cold modified Hank’s solution (137 mM NaCl, 5 mM KCl, 0.7 mM Na_2_HPO_4_, 5 mM glucose, 2.5 mM CaCl_2_, 1.2 mM MgSO_4_, and 4.17 mM NaHCO_3_, pH 7.2). Within 10 min post-dissection, the cerebella were carefully freed from their meninges. Subsequently, 350 µm-thick sagittal slices were prepared using a McIllwain tissue chopper (Föhr Medical Instruments, Egelsbach, Germany). Slices were then transferred into pre-warmed Neurobasal culture medium supplemented with GlutaMax, B27 supplement, and penicillin/streptomycin. They were placed onto 0.4 µm pore-sized membranes (Millicell-CM, Millipore, Bedford, MA) and cultured in 6-well cell culture plates at 37°C, positioned on the interface between 1 ml of culture medium and humidified air with 5% CO_2_. The day of preparation was designated as DIV 0 (Days In Vitro 0).

The culture medium was refreshed every other day. To induce inflammatory conditions, the culture medium was replaced with fresh medium containing either 10 ng/ml (low concentration) or 100 ng/ml (high concentration) of lipopolysaccharide (LPS) and interferon-gamma (IFG) on DIV 3. For control treatments, the medium was replaced by new medium only. Following a six-hour incubation period, the medium was replaced with fresh culture medium without LPS and IFG and incubated for an additional 3 or 7 days for P6 and P0 cultures, respectively. Thereafter, slices were fixed in 4% paraformaldehyde dissolved in phosphate-buffered saline (PBS, pH 7.4) at 37 °C for 30 min.

To block signaling by tumor necrosis factor alpha (TNFa), cultures were incubated simultaneously with LPS/IFG and the TNFa receptor antagonist, R-7050 (Merck KGaA, Darmstadt, Germany) at a final concentration of 2.5 µM. R-7050 was prepared as a 10 mM stock solution in dimethyl sulfoxide (DMSO). For these experiments, a proportional amount of DMSO was added to controls. To deplete microglia from slice cultures, the CSF1R inhibitor PLX3397 (Merck, Darmstadt, Germany) was used at a concentration of 1 µM. A stock solution of PLX3397 was prepared at 10 mM in DMSO and diluted 1:10,000 in neurobasal medium. PLX3397 was administered simultaneously with LPS. While LPS was removed after 6 h, PLX3397 was continuously supplied until DIV 6 for P6 cultures and DIV 10 for P0 cultures. Subsequently, slices were fixed in 4% paraformaldehyde. Again, control cultures were treated with an appropriate concentration of DMSO only (1 µM).

### Immunohistochemistry

Immunostaining was done following previously established protocols [32]. Briefly, fixed slice cultures were subjected to heat treatment at 80 °C for 30 min in 10 mM sodium citrate at pH 7.0 to facilitate antigen retrieval. Subsequently, slices were permeabilized by incubation in 1% Triton X-100/PBS for 30 min. Following several washes in PBS, nonspecific antibody binding sites were blocked for one hour in 0.2% gelatin/PBS. Next, slices were incubated at 4 °C overnight in primary antibodies diluted in 0.2% gelatin/PBS supplemented with 1 mM CaCl_2_ and 0.5 mM MgCl_2_. The primary antibodies utilized included: mouse anti-Calbindin D-28k (Calb1, 1:2000, Sigma, Germany), a polyclonal anti-EN2 serum (En2, 1:500, as described above), and rabbit anti-IBA1 (1:500, Wako Chemicals, United States). After thorough washing, slices were incubated with fluorescently labeled secondary antibodies diluted in 0.2% gelatin/PBS for two hours at room temperature (Alexa goat anti-mouse 488 and Alexa goat anti-rabbit 546, 1:1000, Thermo Fisher Scientific, Germany). Hoechst 33,342 (1 µg/ml in PBS, Thermo Fisher Scientific) was used for nuclear counterstaining for 10 min. Finally, the stained slices were mounted on microscope slides and covered with coverslips using Fluoromount-G (Thermo Fisher Scientific, Germany). The specificity of the CALB1- and IBA1-antibodies used has been extensively documented [43, 44]. The specificity of the En2 antibody generated here is described in the suppl. Figure 1. All staining procedures included controls in which the primary antibody was omitted.

### Microphotography and image processing

All images were captured using an A1R HD25 Ti2E Resonant Laser Scanning microscope (Nikon, Düsseldorf, Germany). Uniform settings, including laser intensity, gain, and offset, were maintained across all imaging sessions. Overview images of the slices were obtained using a 20 × objective (CFI P-Apochromat VC 20x/0.75/1.00), while detailed images of PCs were acquired using a 60 × objective (CFI P-Apo Lambda Oil 60x/1.40/0.13). RGB channels were utilized for image acquisition: Hoechst staining for nuclei (blue), CALB1 for PC morphology (green), and variable red labeling for IBA1 and EN2. The resonant mode was employed to capture 1080 × 1080 RGB z-slices with a slice thickness of 2 µm for 20 × images and 0.5 µm for 60 × images. Only linear adjustments were applied for image presentation using Adobe Photoshop Elements 2022. To isolate individual cells from 3D image stacks, CALB1-positive pixels within the soma of a given cell were identified and joined with all contiguous CALB1-positive pixels. Measurements described below were derived from 3D data. For presentation purposes, cells were projected onto two dimensions using a maximum intensity projection.

### Image analysis

Due to the considerable variation in PC morphology within slice cultures, careful selection criteria were applied to ensure consistency in cell analysis for comparison across groups. Slices containing fewer than ten PCs were excluded from analysis. Only PCs situated in close proximity to other PCs yet exhibiting a clearly distinguishable dendritic tree were considered. Typically, this criterion was met when fewer than 5 cells were clustered together, and only those cells with identifiable entire dendritic trees were selected for analysis. Representative images of PCs shown below for each group accurately depict the characteristics of that group.

Selected PCs were analyzed using Imaris 6.1.4 (Oxford Instruments, United Kingdom). Raw confocal images in nd2 format were imported into Imaris, and z-stacks were reconstructed to create an Imaris Surpass view. PCs of interest were then optically isolated, and the filament tracer tool was employed to semi-automatically trace their dendritic path. The starting point of the stem dendrite was manually set at the soma surface, and each dendritic terminal point was also marked manually. The software automatically tracked dendritic segments in 3D and assigned branching points. Any segments incorrectly identified by the software were manually corrected. Subsequently, the 'center' tool was used to align the overlays of segments and branching points, and the 'smooth' tool was used to refine the generated volumes. Relevant parameters were extracted and processed in R [45], with scripts available upon request from the authors.

In Imaris, a 'filament' refers to the complete dendritic arbor, extending from the soma to all terminal points. A dendritic tree consists of segments separated by branching points, with the total length of a dendrite representing the sum of its segments. Segments are assigned numeric level values, denoting the degree of branching. The initial dendritic segment emanating from the soma is assigned level 1, with subsequent branches receiving incrementally higher-level values. Further details are available in the Imaris Reference Manual version 7.5.

### Western Blotting

To analyze TNFa present in the culture supernatant, 1 ml of culture medium was collected, and the protein content was determined using the PierceTM BCA Protein Assay Kit (Thermofisher, Rockford, USA). The protein concentration was equal in controls and treated cultures. Consequently, 18 µl of supernatant was mixed with 6 µl of 4 × Laemmli buffer (Biorad, Munich, Germany) containing 100 mM dithiothreitol. Samples were then heated to 96 °C for 5 min and finally separated on a gradient SDS gel page (Biorad, Munich Germany). Monomeric TNFa is expected to run at about 17 kDal. After blotting onto a polyvinylidene difluoride membranes, membranes were washed in TBS with 0.1% Tween20 (TBST), incubated in blocking solution containing 5% non-fat dry milk powder in TBST before incubating with TNFa (1:1000, Invitrogen) in blocking buffer at 4°C overnight. After washing, blots were incubated in HRP-linked secondary antibody (1:40,000, Invitrogen) at RT for 1 h and imaged using enhanced chemiluminescent staining (Westar Supernova, Cyanagen, Bologna, Italy). As an additional control for protein concentrations in our samples, we quantified Coomassie blue stained gels, focusing on the albumin present in the B27 medium which by far dominates total protein content. For Coomassie staining, 2 µl of culture supernatant were loaded.

### Statistical analyses

All data processing and statistical analyses were conducted in R [45] using the packages car [46], multcomp [47], emmeans [48], dunn.test [49], and drc [50]. For data analyzed with ANOVA, we initially verified that they met the assumptions for this parametric test [51]. If these assumptions were not met, we applied a square root transformation (for count data, as their variability scaled with the mean) prior to analysis. Data that still did not meet the requirements of parametric tests after transformation were analyzed using Wilcoxon's rank test or the Kruskal–Wallis test followed by Dunn's test for multiple pairwise comparisons. One- and two-way ANOVA and subsequent post hoc testing were conducted, accounting for unbalanced group sizes. Homogeneity of variances across groups was assessed using Levene's test. Post hoc testing for ANOVA was performed using the R package emmeans. P-values for post hoc tests were adjusted using the Benjamini–Hochberg false discovery rate method.

To compare the degree of branching (branch levels), we fitted the number of PCs reaching a given branch level (2, 3, …, 10) to a nonlinear regression model. The relationship between branch levels and Purkinje cell numbers was described using a 4-parameter log-logistic curve (a Hill curve):

$$\# {\text{Pc }}\sim {\text{ c }} + \, \left( {{\text{d}} - {\text{c}}} \right)/\left( {{1 } + {\text{ exp}}\left( {{\text{b}}*\left( {{\text{log}}\left( {\text{x}} \right) \, {-}{\text{ log}}\left( {\text{e}} \right)} \right)} \right)} \right)$$

Here, #Pc denotes the number of PCs, "e" represents the average number of levels reached by the set of PCs under consideration, and "b" indicates the slope around "e". Parameter "d" accounts for the number of PCs in each treatment group. "x" was set to 0, as this parameter signifies the theoretical number of PCs which have an infinite value for branch levels. Curve fitting was performed using the R package drc. Parameters "e" and "b" obtained for wild-type and L7En-2 PCs, as well as following treatment with LPS/IFG, were compared using z-tests implemented in the compParm function of this package. Adjustments for multiple testing were made using the Benjamini–Hochberg procedure.

## Results

### The impact of prolonged EN2 expression in Purkinje cells on their differentiation in vivo is mirrored in vitro

**Fig. 1:**
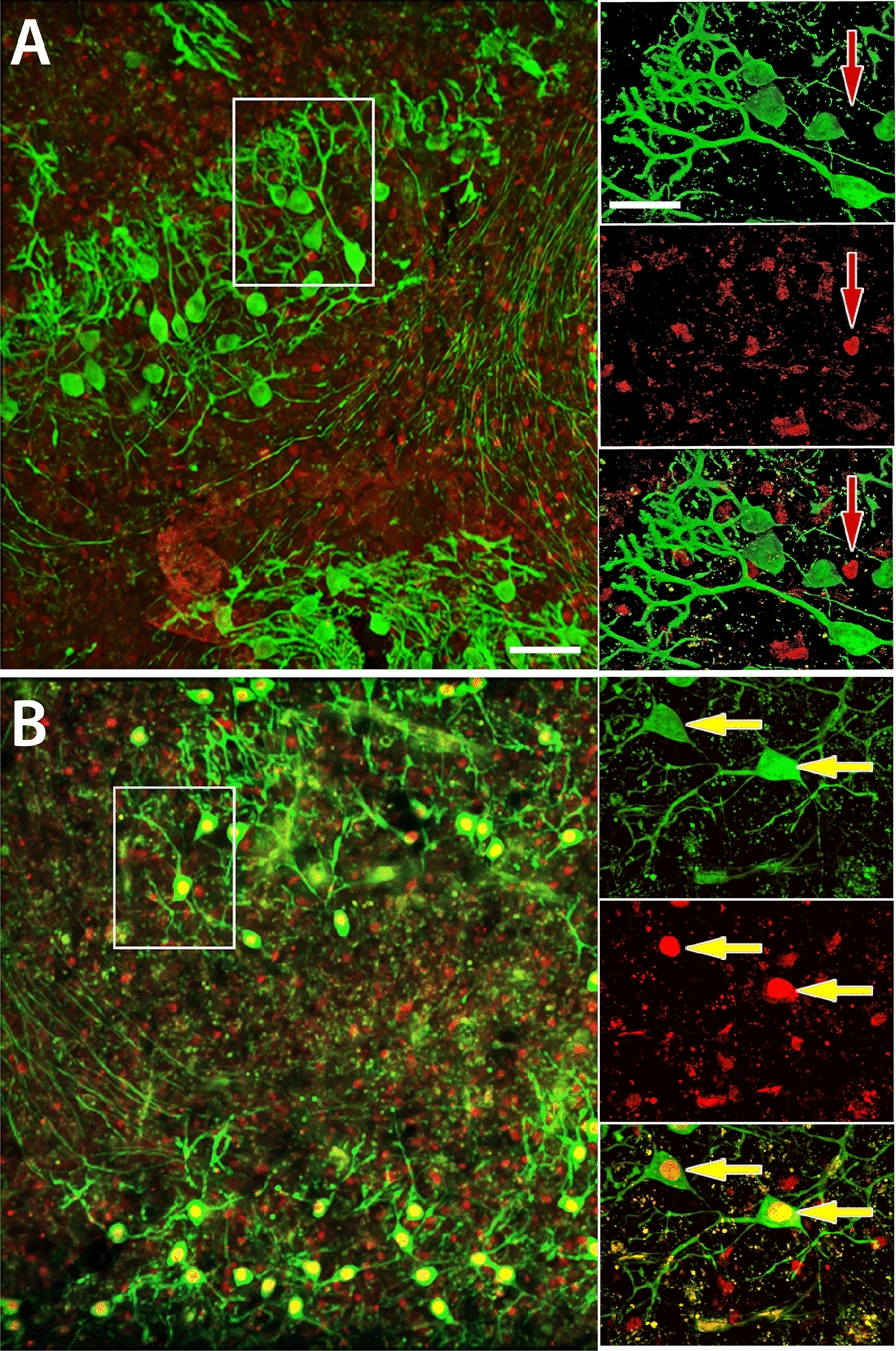
Engrailed-2 overexpression affects dendritogenesis of PCs developing in slice cultures. Slices prepared from 6-day-old mice and cultured for 6 days were immunohistochemically stained for CALB1 (green) and EN2 (red). Note the simple and sparsely ramified dendrites of Purkinje cells of L7En-2 mice (**B**) as compared to cells derived from FVB/N wildtype mice (**A**). Staining for EN2 (red) reveals overexpression of this transcription factor in Purkinje cells of L7En-2 derived slices (red arrow). Endogenous EN2 expression in numerous non-Purkinje cells is visible in cultures from both genotypes. Bar is 100 µm for overviews and 25 µm for inserts

To investigate whether prolonged expression of the homeobox transcription factor Engrailed-2 (EN2) in Purkinje cells (PCs) interacts with inflammatory stimulation in slice cultures, we first confirmed that the typical reduction in cerebellar PC dendritogenesis observed in intact mice (L7En-2 mice; [30, 32]) could also be replicated in vitro. As illustrated in Fig. 1, the distinctive dendritic morphology of EN2-overexpressing PCs in slice cultures clearly contrasts with the well-developed PC dendritic trees observed in slices from FVB/N wildtype mice. Additionally, as depicted in Fig. 1, PCs in slices from L7En-2 animals exhibit strong expression of EN2, unlike those from wildtype mice. This demonstrates that essential aspects of EN2 overexpression on PCs dendritogenesis in vivo are faithfully repeated in vitro.

### Lipopolysaccharides and interferon-gamma reduce PC dendritogenesis in cerebellar slice cultures of P6 wildtype and L7En-2 mice

Lipopolysaccharides (LPS) and interferon-gamma (IFG) are commonly utilized to induce inflammation both in vivo and in vitro at concentrations ranging from 10 to 100 ng/ml [52–54]. In a first set of experiments, we applied 10 or 100 ng/ml of LPS/IFG to cerebellar slice cultures obtained from 6-day-old FVB/N pups on day 3 in vitro. After 6 h of stimulation, the stimulus was removed, and the cultures were maintained under standard incubation conditions for another 3 days in vitro.

**Fig. 2:**
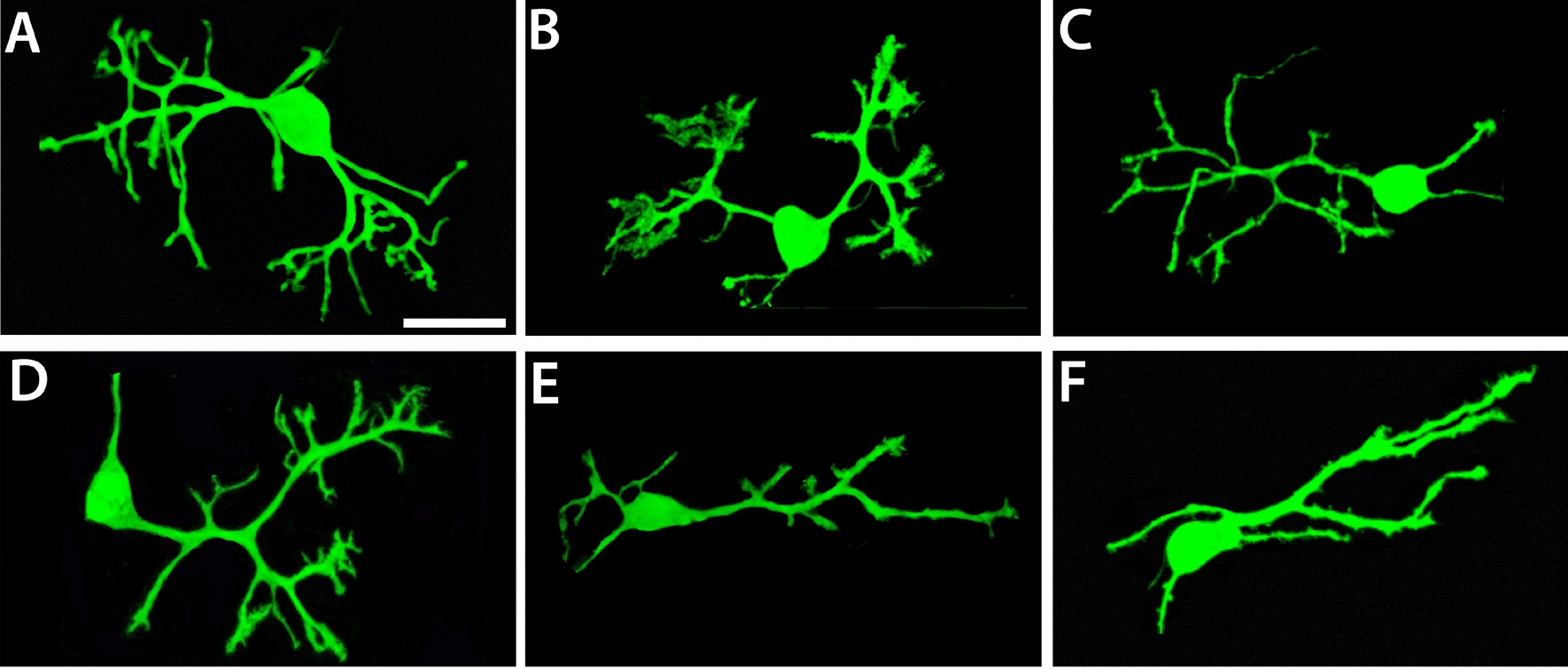
Effect of different concentrations of LPS/IFG on PC arborization in wildtype and L7En-2 PCs. Cerebellar slice cultures were prepared from 6-day-old FVB/N wildtype (**A**–**C**) or L7En-2^tg^ transgenic mice (**D**, **E**), treated with either low (lc, 10 ng/ml in **B**, **E**) or high concentration (hc, 100 ng/ml in **C**, **F**) of each LPS and IFG and fixed at DIV 6. In **A** and **D**, typical untreated PCs of either genotype are shown. In both genotypes, the overall complexity of the dendritic tree seems to be diminished by inflammation

Upon microscopic examination, PCs in culture typically exhibited one, occasionally two or three repeatedly branched dendrites. Initial observations of LPS/IFG-treated PCs indicated a noticeable effect on the morphology of both wildtype and L7En-2 PCs, which showed reduced dendrite size and branching (Fig. 2). For quantitative morphometric analysis we focused on the one dendrite of each PC having the greatest total length, as it best reflects developmental polarization of PCs [19].

**Fig. 3:**
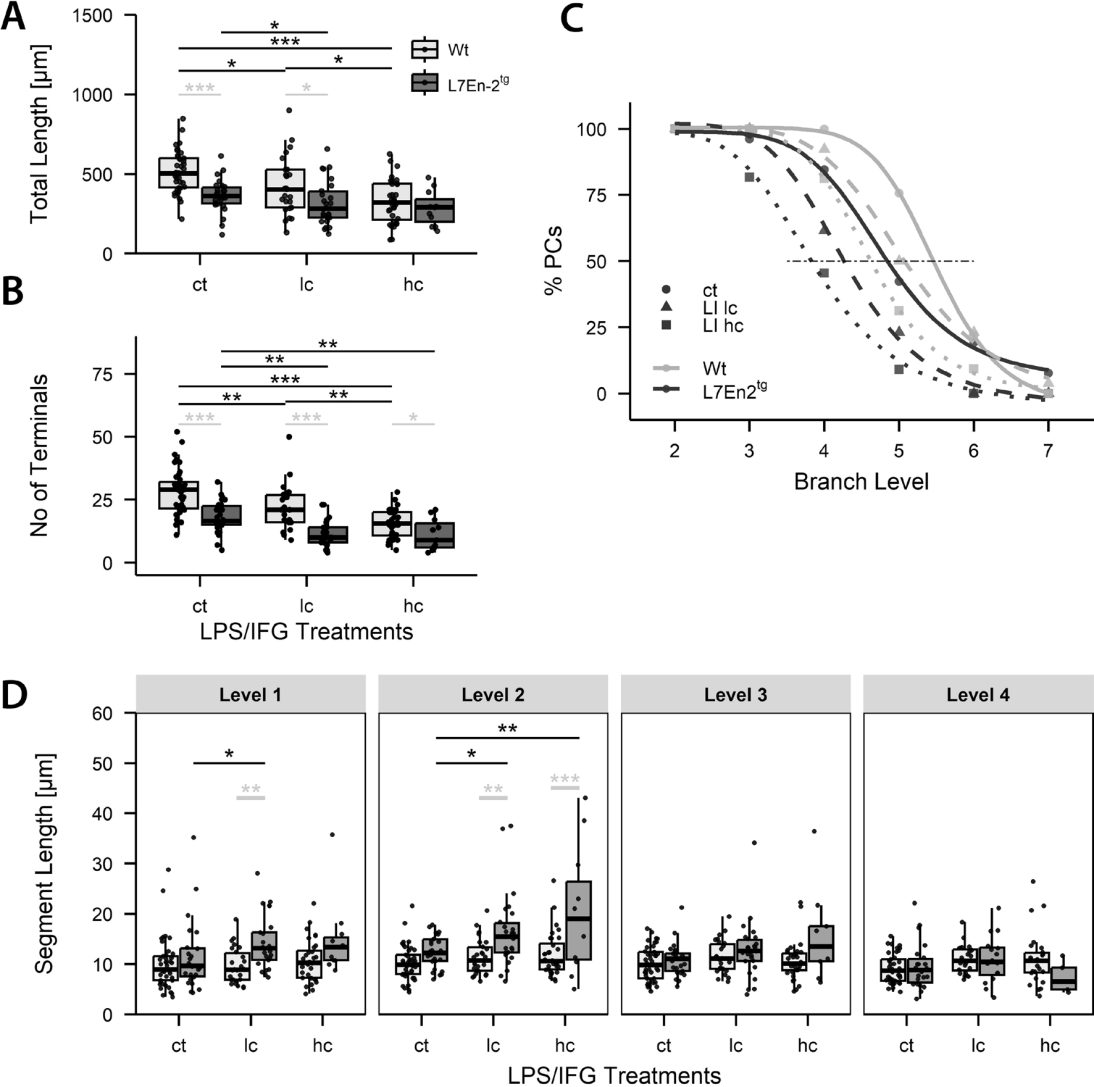
Additive effects of LPS/IFG treatment and EN2 overexpression on PC dendritogenesis. Cerebellar slice cultures prepared from 6-day-old FVB/N wildtype (**A**–**C**) or L7En-2^tg^ transgenic mice were treated with low (lc) or high (hc) concentrations of LPS/IFG and analyzed at DIV 6. Both genotype and inflammatory stimulation reduced total dendritic length (**A**) and number of dendritic terminals (**B**). Consistently, numbers of PC dendrites reaching a higher branch levels are reduced by EN2 expression and inflammatory stimulation (**C**). This may be taken from the left shift of the Hill curves describing the distribution of segment levels due to inflammatory stimulation (for wildtype, p_ct,lc_, p_ct,hc_, p_lc,hc_, all < 0.002; for L7En-2, p_ct,lc_, p_ct,hc_, p_lc,hc_, all < 0.02) and to the genotype. Genotype effects were assessed at identical treatment levels (p_ct_, p_LI lc_, p_LIhc_, all < 0.0003). To facilitate comparison, PC numbers are given as percentages of the total number of PCs analyzed. Statistics were calculated on original values. Segment lengths of wildtype PC dendrites were similar across levels and not influenced by inflammatory stimulation (D; all p-values larger than 0.1). In L7En-2 PCs, inflammation increased segment lengths at levels 1, where the low dose of LPS resulted in a significant increase (p_ct,lc_ = 0.0236). The same was true for level 2 (p_ct,lc_ = 0.0191). An even further increase was seen with high dose of LPS (p_ct,hc,_ = 0.0021). At level 3 the change approached the threshold of significance (p_ct,hc_ = 0.0682) in tg PCs. No differences were seen at level 4. Significance levels for genotype differences are indicated by light gray symbols, and those for differences due to LPS/IGF-treatment are indicated by black symbols). Two-way ANOVA *p < 0.05, **p < 0.01, ***p < 0.001; n_ct, wt_ = 41, n_lc, wt_ = 26, n_hc, wt_ = 32, n_ct, tg,_ = 26, n_lc, tg_ = 26, n_hc, tg_ = 11)

Figure 3 summarizes the quantitative morphological differences between dendrites of FVB/N wildtype and L7En-2 transgenic PCs grown in slice cultures under control conditions and following treatment with LPS/IFG. Under control conditions, L7En-2 PCs exhibited significantly shorter total dendrite lengths, lower number of terminals, and lower maximum branch levels as compared to FVB/N wildtype cells (Fig. 3A–C). As outlined above, EN2 overexpression reduced PC dendritogenesis.

In cerebellar slices from FVB/N wildtype mice, treatment with LPS/IFG led to significant and dose-dependent reductions in total dendritic length, maximum branch level, and the number of dendritic terminals (Fig. 3A–C). These findings suggest that inflammation adversely affects dendritic branching and process outgrowth. Similarly, in slices from mice with prolonged expression of EN2 in PCs, LPS/IFG treatment resulted in a slight but still significant reduction in total dendritic length. Additionally, as observed in slices from FVB/N wildtype animals, the maximum branch level and the number of terminals were robustly reduced in L7En-2 PCs (Fig. 3A–C). This indicates that the already shorter and less complex dendrites of EN2-overexpressing PCs were further diminished by inflammatory stimulation.

Both prolonged expression of EN2 and treatment with LPS/IFG led to reductions in total dendritic length, yet different levels of dendritic segments were variably affected under these conditions (Fig. 3D). In wildtype PCs, the average segment length remained relatively constant across levels 1 to 4, showing minimal response to LPS/IFG treatments. In contrast, in EN2-overexpressing PCs, segment lengths at levels 1 and 2 were notably influenced by inflammatory stimulation, exhibiting an increase in average segment length compared to FVB/N wildtype PCs. This trend continued at level 3, where mean segment lengths tended to increase after inflammatory stimulation (p = 0.068), contrasting with the lack of significant effect observed in FVB/N wildtype PCs (p = 0.278). At level 4, segment lengths were comparable between FVB/N wildtype and L7En-2 PCs and remained unaffected by inflammation. It is worth noting that segments at level 4 were particularly sparse, especially in treated L7En-2 PCs (Fig. 3C).

The aforementioned analyses demonstrate that dendritic development in FVB/N wildtype and L7En-2 overexpressing PCs in slice cultures from early postnatal murine cerebella is influenced by inflammatory stimulation. Treatment with LPS/IFG affects dendritogenesis in both genotypes. However, the data also allow us to examine whether the overexpression of EN2, which itself affects PC dendritogenesis, sensitizes or desensitizes dendritogenesis towards inflammatory stimuli. Formally, this amounts to asking whether there is a (statistical) interaction between genotype and inflammatory stimulation. We did not observe a statistical interaction for any of the parameters tested (p_interaction_ (total length) = 0.191; p_interaction_ (number of terminals) = 0.267; p_interaction_ (segment length at all levels) between 0.12 and 0.19). Thus, the effects of EN2 expression and inflammation, although independent, appear to be additive.

### Microglia mediates inflammation induced Purkinje cell deficiencies

**Fig. 4:**
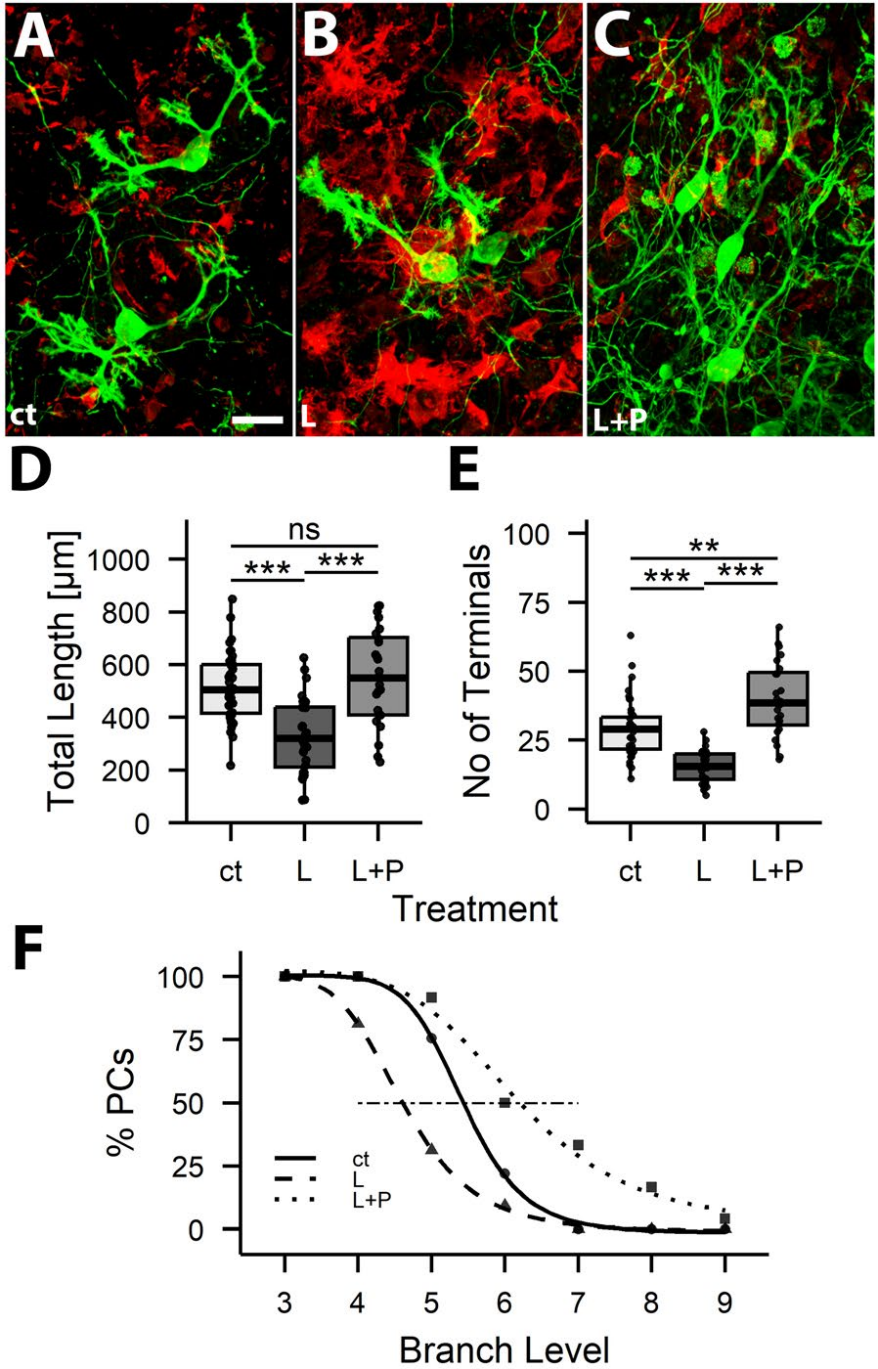
Blocking LPS-induced microglia proliferation restores PC dendritogenesis. Cerebellar slice cultures prepared from 6-day-old C57Bl6/J mice were treated with LPS (L) and PLX3397 (P) and fixed at DIV 6. Morphometric analysis revealed a clear difference in total dendritic length, number of branch levels and number of terminals between LPS and untreated cultures (ct), and also between LPS- and PLX-treated (L + P) vs. LPS treated (L) cultures. Overall, parameters of ct and L + P cultures were rather more similar to each other than to those of LPS-treated cells (“ns” not significant, ** p < 0.01, *** p < 0.001 (n_ct_ = 41, n_L_ = 32, n_L+P_ = 24)

In the course of the previous experiments we realized that the dendritic arborization was mainly affected by LPS, and little, if at all, by IFG (see Fig. 4 and compare to Fig. 2C and F). Therefore, we treated slice cultures in the following experiments with LPS alone.

Inflammation in brain tissue is considered to be mediated by microglia. To probe whether microglia is involved in the inflammatory responses of PCs described above, we blocked LPS induced microglia proliferation by simultaneous application of LPS and PLX3397 (Pexidartinib; PLX for short) to cerebellar slice cultures derived from 6-day-old C57Bl6/J pups. PC morphology was analyzed three days thereafter.

As expected, LPS treatment caused a visible increase in IBA1-positive microglial cells in slice cultures. This increase in microglia numbers was blocked by additional PLX treatment (Fig. 4A–C). Thus, the fraction of IBA1-positive pixels in randomly selected regions of interest in control cultures was 0.15 +/− 0.15; in LPS treated cultures it was 0.47 +/− 0.24 and in LPS + PLX treated ones it was 0.11 +/− 0.07 (n = 9 regions of interest). As described above, LPS treatment of cerebellar slice cultures caused a significant reduction in total dendritic length and a reduction in branching of PC dendrites also in this experimental series (Fig. 4D–F). When PLX was added to LPS treated cultures, LPS induced effects were abolished, and indeed, dendrites in LPS + PLX treated cultures were somewhat more complex than in control cultures.

### Inflammation affects dendritogenesis in an age-dependent manner

**Fig. 5:**
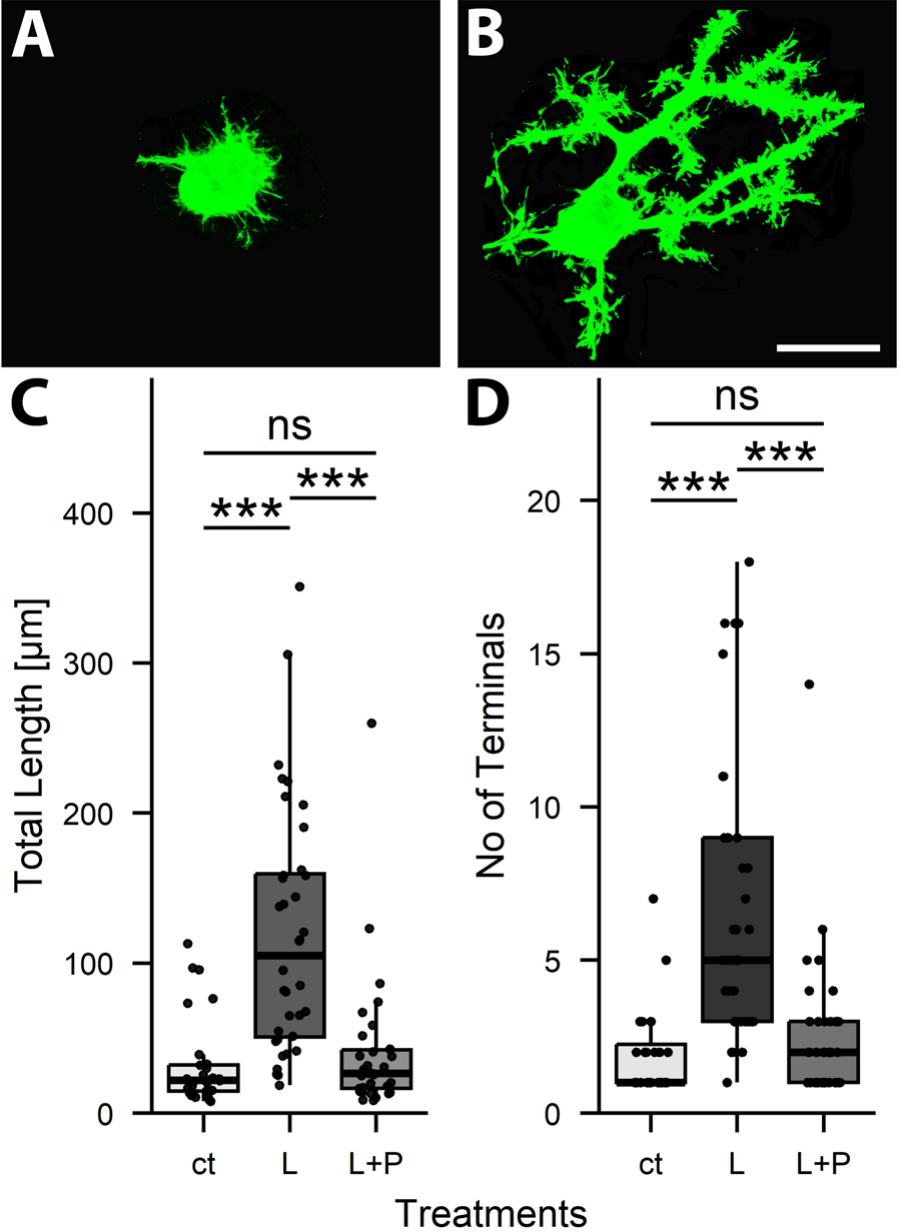
Inflammation enhances PC dendritogenesis in slice cultures derived from newborn mice. Cerebellar slice cultures were prepared from newborn C57Bl6/J wildtype mice, treated with LPS (L) or LPS and PLX (L + P) and fixed at DIV 10 (bar is 20 µm). Control cultures (ct) were not treated. A visual comparison already demonstrated clear differences in PC arborization between untreated (**A**) and LPS treated PCs (**B**). Morphometric analysis revealed a clear difference in total dendritic length (**C**), and in the number of terminals (**D**) between untreated and LPS treated PCs as well as between LPS and LPS + PLX treated PCs. (“ns” not significant, ***p < 0.001; n_ct,_ = 25, n_L_ = 37, n _L+P_ = 34)

Prior to the elaboration of their definitive dendrites described above, Purkinje cells transiently display a bipolar shape and acquire a stellate shape with disoriented dendrites. This transitory phase starts perinatally, at the end of PC migration, and lasts up to postnatal day 6 [19]. To test whether this transitory phase is also sensitive to inflammatory stimuli, we prepared cerebellar slice cultures from newborn C57Bl6/J pups and treated them with LPS and/or PLX3397 for 6 h at DIV 3. Thereafter, the medium was removed and replaced with control medium, or medium containing PLX3397. Slices were fixed at DIV 10, and PC morphology was analyzed as above. As expected, PCs derived from P0 cultures were much less developed than those of P6 cultures and showed the typical multipolar appearance of PCs characteristic at this developmental stage [19]. Their processes were about 10 µm in length, did not branch and thus had only one terminal point. LPS treatment reduced the numbers of these primitive dendrites, but also caused a significant elongation of the remaining dendrites (to a length of about 50 µm on average). In addition, dendrites in LPS treated slices from P0 mice were more branched than in controls and thus had higher numbers of terminal points (Fig. 5). Blockade of microglia proliferation with PLX3359 (PLX) completely abrogated these effects of LPS.

### Tumor necrosis factor alpha signalling affects Purkinje cell differentiation

**Fig. 6:**
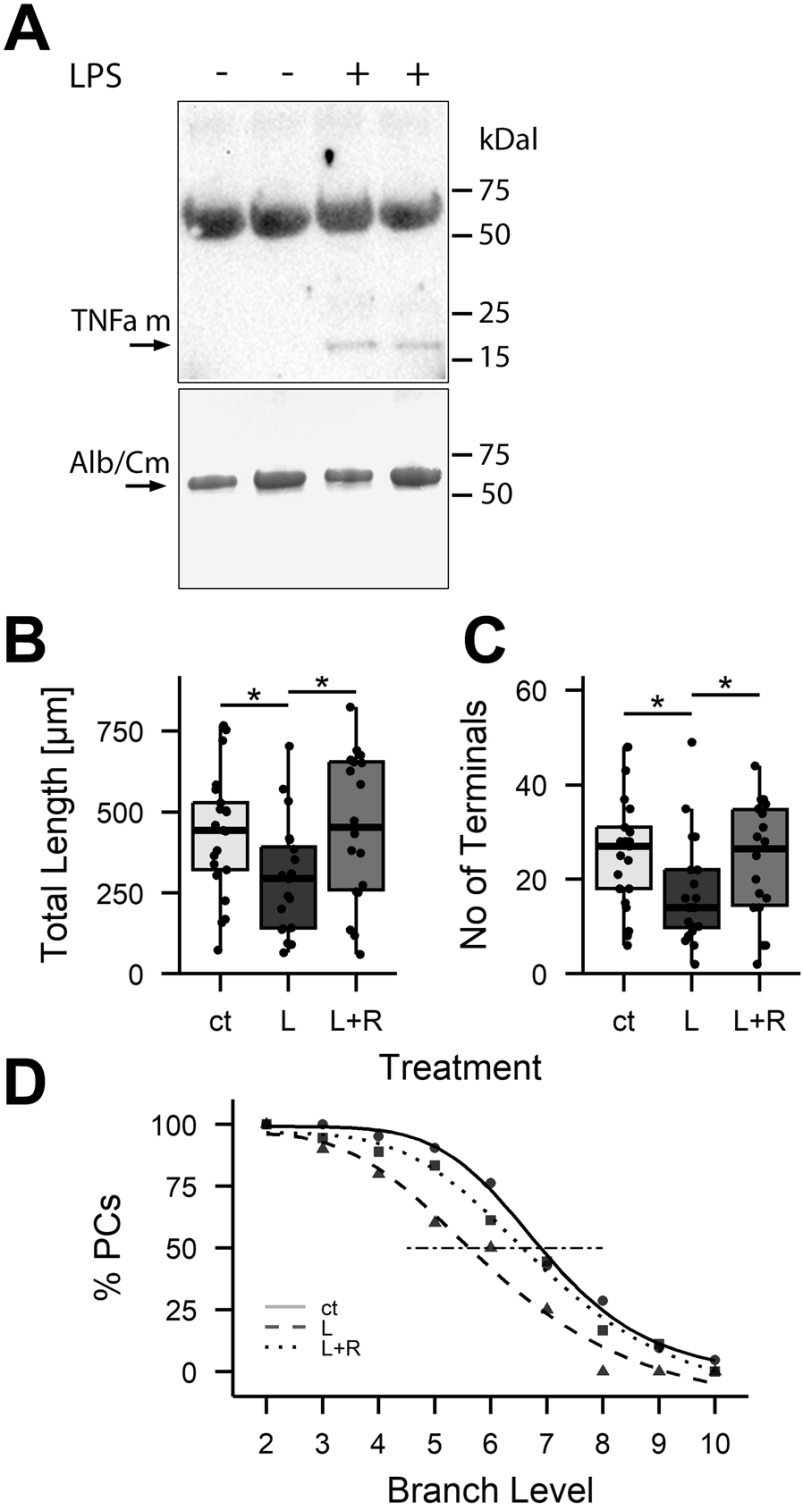
TNF alpha receptor blockage inhibits LPS mediated reduction of PC arborization in P6 cultures. Slice cultures prepared from 6-day-old C57Bl6/J wildtype mice were treated with LPS (L) and R-7050 (R) to block TNFaR signaling. As revealed by Western blotting, the supernatant of LPS treated cultures contained increased levels of monomeric TNFa (**A**). The band at about 60–70 kDal probably reflects weak non-specific binding of TNFa antibody to albumin and maybe used as a loading control. Albumin is quantitatively the predominant protein in the culture medium, and the lower panel shows albumin levels as assessed by Commassie staining (Alb/Cm). Total dendritic length, number of terminals and dendritic branching of LPS treated PCs were significantly different from control and LPS and R-7050 (L + R) treated cells (*p < 0.05). In contrast, controls and L + R treated cells could not be distinguished (all p > 0.28). n_ct,,_ = 21, n_L_ = 20, n_L+R_ = 18

TNF-alpha (TNFa) is a pro-inflammatory cytokine known to be upregulated in stimulated microglia [55]. Consistently, in cultures derived from 6-day-old C57Bl6/J wildtype mice, treatment with LPS not only prominently increased numbers of microglia (Fig. 4), it also increased the concentration of TNFa in the culture supernatant (Fig. 6A). We therefore probed the role of TNFa signaling for the inflammatory effects on dendritogenesis described above.

To do so, we used R-7050 to block TNFa receptor activation. Since R-7050 has to be diluted in DMSO, an agent well known to affect cellular differentiation in a dose dependent manner [56, 57], controls were supplemented with the same concentration of DMSO as needed to apply R-7050 (i.e., 0.1% v/v). In initial experiments, we had ascertained that this concentration of DMSO per se had at best a minimal effect on PC dendritogenesis.

As described above for cultures derived from P6 animals, LPS significantly reduced the total dendritic length, the number of dendritic terminals and the maximal branch level achieved by wildtype PCs also in this series of experiments. All these effects were efficiently blocked by 2.5 µM of the TNFa receptor blocker R-7050 (Fig. 6B–D).

**Fig. 7:**
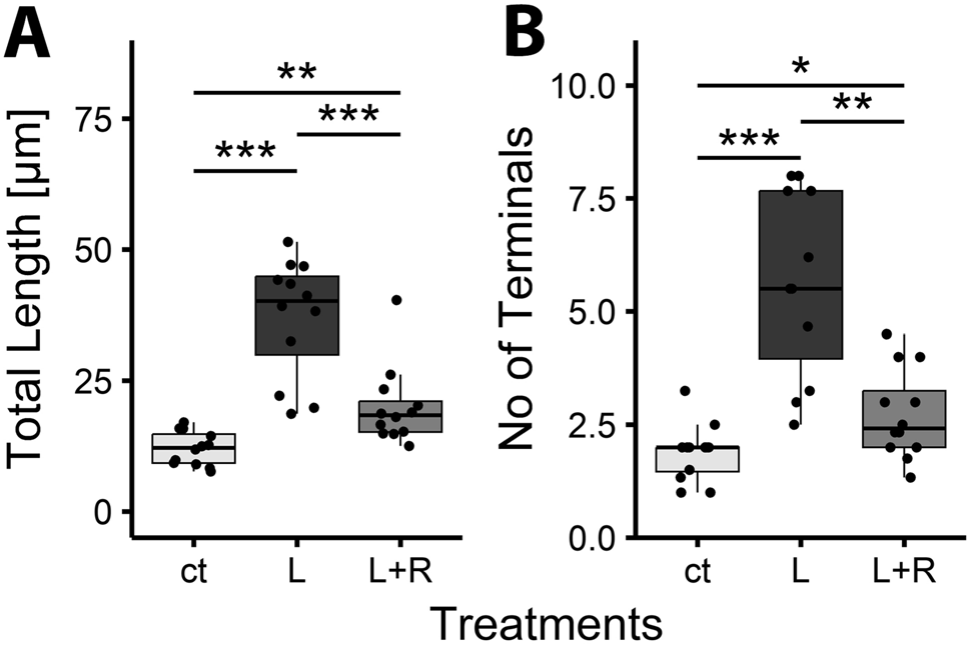
Inhibition of LPS induced effects on PC dendritogenesis by TNFaR blocker R-7050. Cerebellar slice cultures prepared from newborn C57Bl6/J mice were treated with LPS (L) or LPS and R-7050 (L + R) and fixed at DIV 10. Morphometric analysis revealed a clear difference in total dendritic length (**A**), and in the number of terminals (**B**) between untreated (ct) and LPS-treated PCs as well as between LPS- and LPS/R-7050-treated PCs. (*p < 0.05, **p < 0.01, ***p < 0.001; n_ct,,_ = 12, n_L_ = 12, n_L+R_ = 12)

Similarly, the effects of LPS on dendritogenesis of PCs derived from neonatal (P0) mice were abrogated by blocking TNFa signaling with R-7050 (Fig. 7). As described above, in these cultures, LPS increased total dendritic length and the number of terminals per cell. When concomitantly treated with R-7050, all these LPS induced effects were strongly attenuated (Fig. 7).

## Discussion

The present findings demonstrate the effectiveness of slice cultures for evaluating the impact of prolonged Engrailed-2 expression on Purkinje cell dendritogenesis, aligning with previous research [32]. Additionally, they highlight the influence of inflammatory stimuli on dendritogenesis, which resulted in a Purkinje cell phenotype akin to that induced by Engrailed-2. Crucially, these effects were found to be cumulative. The inflammatory effects were contingent upon microglia proliferation and susceptible to inhibition of TNF alpha receptor (TNFaR) signaling. Finally, we provide evidence suggesting that the morphogenetic consequences of inflammation hinge on the developmental stage at which Purkinje cells are affected.

### Effect of protracted expression of Engrailed-2 and of inflammation on dendritogenesis

Our data from P6 slice cultures reveal a reduction in PC dendritogenesis when EN2 expression extends beyond the physiological period, mirroring previous observations in intact mice [30, 32]. A strikingly similar effect on dendritogenesis was observed in wildtype PCs exposed to inflammatory signals. We evaluated four parameters—total length of the main dendrite, segment lengths, branch level, and total number of terminals—to provide a detailed quantitative description of dendritic morphology. All these parameters were affected in a consistent manner by prolonged EN2 expression and inflammation. While EN2-expressing Purkinje cells and those subjected to inflammatory stimuli were distinguishable from wildtype control cells, the difference between L7En-2 and LPS-treated wildtype cells was minimal. This was evident in micrographs of individual cells (see, e.g., micrographs in Figs. 1A, C and 2) and supported by statistical parameters presented in Fig. 3. The morphogenetic effects of prolonged EN2 expression and inflammatory stimulation suggest that these interventions converge, ultimately resulting in an additive impact on PC dendritic growth and branching. Such alterations may have implications for synaptic integration by PCs (e.g. [58–60].

In fact, synaptic proteins, axon guidance molecules, and adhesion contacts are dysregulated in L7En2 mice [61]. The additive nature of both effects also suggests that neither prolonged expression of EN2 nor LPS treatment, as examined here, represent maximal stimuli for ultimate signal integration in dendritogenesis. While the intracellular signal processing and integration mechanisms of EN2 overexpression and inflammation require further elucidation, our data shed light on how LPS signaling is mediated to PCs. Specifically, the effects of LPS critically rely on the activation and proliferation of resident microglia, as well as TNFa signaling.

### Microglia mediated attenuation of PC dendritogenesis

Microglia and astrocytes are considered to be major mediators in CNS inflammation (see e.g. [62]). In line with this, our findings demonstrate a significant increase in microglia cell numbers, accompanied by enhanced TNFa expression, following treatment with LPS/IFG (or LPS alone). Restoration of PC dendritogenesis was observed upon blocking either microglia proliferation using PLX3397 or TNFa receptor signaling. The somewhat more complex dendritic morphology of LPS + PLX treated PCs as compared to controls probably reflects that PLX also suppresses the microglia activation inevitably resulting from the cultivation procedure.

PLX3397 has been effectively employed to block CSFR1 receptor, thereby inhibiting microglia proliferation and eliminating these cells from brain tissue ([63, 64], and Fig. 4). However, in the developing cerebellum, two additional high affinity targets of PLX3397 are expressed, the KIT receptor in cerebellar inhibitory interneurons and the FLT3 receptor in PCs [65, 66]. While the involvement of the KIT/KITL pathway in PC morphogenesis seems improbable, given the lack of observed morphological, functional, or behavioral phenotypes in KIT ligand-deficient mice (e.g., [67, 68]), Flt3 has been implicated in neuronal stem cell differentiation [69]. In vitro studies have also shown that (activated) microglia express the FLT3 ligand, Flt3l [70]. Thus, PLX3397 might well inhibit microglia-to-PC signaling by blocking Flt3 signaling on PCs. This is all the more true as the concentration of PLX3397 used in our study (1 µM) should not only effectively block the Csf1-receptor (IC50 = 13 nM, but also FLT3 (IC50, 160 nM) [71]. One conundrum, when appraising potential signaling via the FLt3 receptor, is that this receptor is located cytoplasmatically in PCs, rather than at its surface [72]. However, should the Flt3 receptor be involved in the effects observed, this underlines the significance of microglia, which are the sole source of the Flt3 ligand in the CNS. Still further support for the essential role of microglia in inflammation-associated alterations of PC differentiation comes from results obtained with R-7050.

### Microglia effects are dependent on TNFa receptor signaling

R-7050 suppresses TNFa receptor-mediated signaling as well as IL-1beta signal transduction [73]. The IC50 for these two receptors differ only by a factor of 2.3. At the concentration used in our study, both receptors are efficiently blocked. Microglia are sensitive to TNFa and are also a significant source of this cytokine [74–78] (see Fig. 6A), as well as of IL-1beta [79], which integrates microglial effects on neural cells, notably activated astroglia (e.g. [80–82]). Blocking the receptors of these cytokines with R-7050 effectively suppressed LPS-induced effects on dendritic morphology of PCs. This underscores the critical significance of microglial activation for the aberrant Purkinje cell dendritogenesis observed following LPS/IFN treatment.

Intriguingly, TNFa-induced (auto-) stimulation of microglia has been reported to lead to glutamate release from microglia [76], induce glutamate release by Bergmann glia [83], and result in Purkinje cell hyperexcitability [82]. Conversely, IL-1beta has been reported to downregulate glial glutamate receptors at PC synapses [79]. This suggests a mechanistic pathway through which inflammation might interfere and converge with the well-established role of excitatory (synaptic) input for Purkinje cell dendritogenesis (e.g., [84, 85]). Interestingly, microglia-mediated axonal pruning has also been observed to be dependent on neural activity [86].

### Age-dependent influence of inflammation on PC dendritogenesis

Whereas LPS/IFN reduced dendritic expansion in Purkinje cells cultured at P6, it strikingly enhanced dendritic development in cultures derived from newborn (P0) donors. It is well established that brain microglia continue differentiating perinatally and in the juvenile period [87–90]. Available data do not exactly bracket the first postnatal week and do not allow deriving a signature for functional differences between microglial cells, or their reactivity, at P0 and P6. Still, it stands to reason that perinatal microglia differentiation might contribute to the differential effects of inflammation on PC dendrite development in P0 and P6 cultures.

Developmental changes of cerebellar neurons over the first postnatal week are far better understood than that of cerebellar microglia. Between P0 and P6, PCs and indeed all its interacting cells in the cerebellar anlage in situ continue to migrate, differentiate and become increasingly integrated. Not all of these developments may be faithfully recapitulated in slice culture. Most obviously, culturing eliminates input via mossy and climbing fibers. The developmental schedule of these excitatory fiber systems [91–93] suggests that PCs cultured at P6 should differ from those cultured at P0 by having been exposed to extracerebellar input, which is questionable, or at best minimal, before birth.

Numbers of granule neurons and molecular layer inhibitory interneurons (i.e., basket and stellate cells) which both provide direct synaptic input to mature PCs also change dramatically over the first postnatal week. Thus, less than 5% of all cerebellar inhibitory interneurons have formed at P0, and none of these has reached the PC layer, let alone the nascent molecular layer. In contrast, by P6, more than 50% of all inhibitory interneurons have formed, and many of these have reached the by now clearly discernible molecular layer [94, 95]. Also, at P0, proliferating granule cell precursors are limited to a rather thin external granule cell layer; at P6, about 5% of granule cell precursors have formed, and some of these have reached the nascent (internal) granule cell layer [96–98]. Lastly, it may be mentioned that macroglia cells expressing GFAP or MBP are also essentially absent from the nascent white matter at P0, but are well visible there at P6 [99].

Thus, inflammation-stimulated microglia in cultures of neonatal (P0) and P6 cerebella actually operate in quite distinct cellular environments, and may or may not interact with cells that themselves impinge on PC dendritogenesis [84, 100–102]. Untangling and understanding the cellular and molecular basis of these age-specific effects should also be important to tailor potential anti-inflammatory therapies to the developmental of affected individuals. Inflammation during early life has been reported to cause a retardation of dendritic spine formation, abnormal social behavior and depression during adolescence in man [103, 104]. To the best of our knowledge, a systematic correlation between the time course of inflammation and subsequent deficits is not available. A glutamate-dependent mechanism, as mentioned above, is an intriguing candidate to explain age dependent effects of inflammation. Of note, expression of NMDA-receptors, known to impinge on Purkinje cell dendritogenesis, changes dynamically during the early postnatal period [105–108]. To follow up on this issue, model systems which allow cell type specific manipulation of glutamate signaling will be necessary.

## Conclusion

Our studies document that two conditions previously associated with the development of an Autistic phenotype, i.e. EN2 expression and inflammation, converge to affect proper PC differentiation. Given the documented involvement of the cerebellum in the development of Autism and related conditions, the results presented suggest how this model system might be used to dissect the cellular integration of their multifactorial genesis. An unexpected but intriguing finding was that inflammatory stimulation resulted in almost inverse/opposite effects on dendritogenesis in newborn versus 6-day old PCs. Unraveling the basis of this age effect is of interest not least because it might help to devise developmental stage specific, targeted approaches to attenuate inflammation related dysfunction.

## Supplementary Information

Additional file 1. **Suppl. Fig. 1:** Specificity of the En2 antibody. In Western blotting (**A**) of cerebella of wildtype mice (FVB/N and C57Bl6/J), the antiserum strongly stains a single band at 40 kDal which is expected given the calculated molecular weight of En2 (marked by an arrow). This band is nearly absent in cerebellar tissue derived from En2ntd/ntd knockout mice [42]. The fact that the En2 band appears not to be more intense in tissue of mice overexpressing En2 specifically in Purkinje cells (L7En-2 mice) may be rationalized considering that En2 positive granule cells and interneurons outnumber Purkinje cells by a factor of more than 250 [109]. In sections of wildtype cerebella (FVB/N is shown here), antibody En2 intensely stains nuclei in the granule cell and molecular layers but not those of PCs (**B**). Staining of knockout mice (En-2ntd/ntd) did not result in a specific staining, but only in a diffuse background visible only after enhancing brightness (**C**). Staining of cerebellar L7En-2 tissue revealed an intense staining of PC nuclei (**D**), in addition to the staining seen in wildtype tissue. In conjunction with the Western blot results this indicates that the En2 antibody recognizes En2 and not any En2 downstream targets. Bar in B is 50 μm.

## Data Availability

No datasets were generated or analysed during the current study.

## Abbreviations

ASD: Autism spectrum disorder
CNS: Central nervous system
Ct: Control
DIV: Days in vitro
DMSO: Dimethyl sulfoxide
En2: Engrailed-2
IFG: Interferon gamma
LPS: Lipopolysaccharide
PC: Purkinje cell
Tg: Transgenic
TNFa: Tumor necrosis factor alpha
TNFaR: Tumor necrosis factor alpha receptor
Wt: Wildtype
