# Supplementary figures and images for: Engrailed-2 and inflammation convergently and independently impinge on cerebellar Purkinje cell differentiation
Source: J Neuroinflammation. 2024 Nov 28;21:306. doi: 10.1186/s12974-024-03301-6 (PMC11603920; doi:10.1186/s12974-024-03301-6)

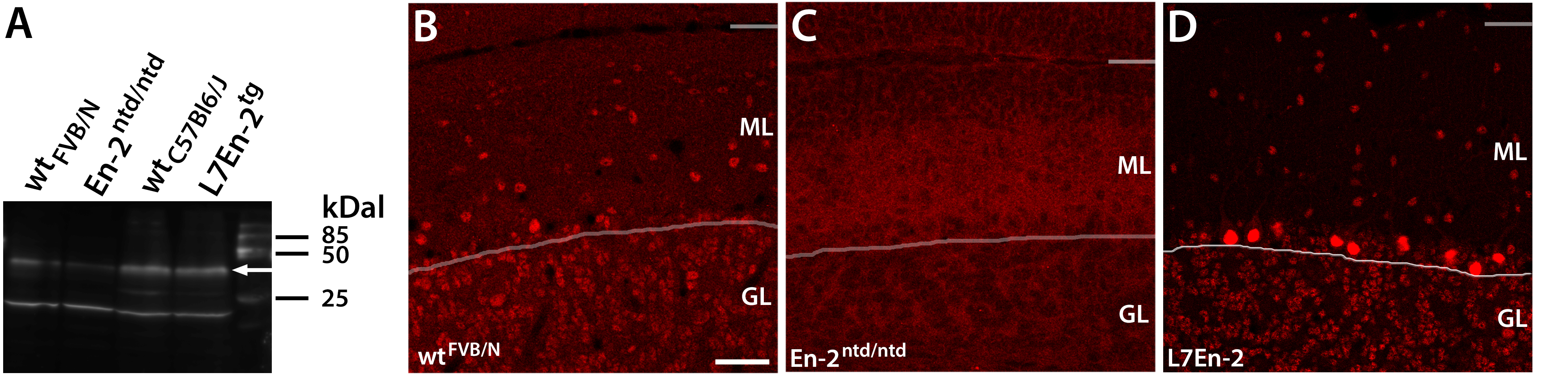

Supplement: Supplementary file 1 — Additional file 1. Suppl. Fig. 1: Specificity of the En2 antibody. In Western blotting (A) of cerebella of wildtype mice (FVB/N and C57Bl6/J), the antiserum strongly stains a single band at 40 kDal which is expected given the calculated molecular weight of En2 (marked by an arrow). This band is nearly absent in cerebellar tissue derived from En2ntd/ntd knockout mice [42]. The fact that the En2 band appears not to be more intense in tissue of mice overexpressing En2 specifically in Purkinje cells (L7En-2 mice) may be rationalized considering that En2 positive granule cells and interneurons outnumber Purkinje cells by a factor of more than 250 [109]. In sections of wildtype cerebella (FVB/N is shown here), antibody En2 intensely stains nuclei in the granule cell and molecular layers but not those of PCs (B). Staining of knockout mice (En-2ntd/ntd) did not result in a specific staining, but only in a diffuse background visible only after enhancing brightness (C). Staining of cerebellar L7En-2 tissue revealed an intense staining of PC nuclei (D), in addition to the staining seen in wildtype tissue. In conjunction with the Western blot results this indicates that the En2 antibody recognizes En2 and not any En2 downstream targets. Bar in B is 50 μm. [file 12974_2024_3301_MOESM1_ESM.jpg]
